# Optimization of Ultrasound-Assisted Extraction of Dietary Fiber from Yellow Dragon Fruit Peels and Its Application in Low-Fat Alpaca-Based Sausages

**DOI:** 10.3390/foods12152945

**Published:** 2023-08-03

**Authors:** Wilber Vilcapoma, Johannes de Bruijn, Carlos Elías-Peñafiel, Clara Espinoza, Lucero Farfán-Rodríguez, Jorge López, Christian R. Encina-Zelada

**Affiliations:** 1 Departamento de Tecnología de Alimentos y Productos Agropecuarios, Facultad de Industrias Alimentarias, Universidad Nacional Agraria La Molina, Av. La Molina s/n Lima 12, Lima 15024, Peru; 2 Departamento de Agroindustrias, Universidad de Concepción, Av. Vicente Méndez, n°595, Chillán 3812120, Chile; 3 Departamento de Tecnología de Alimentos, Universidad Nacional del Centro del Perú, Huancayo 12006, Peru; 4 Departamento de Ingeniería Química, Facultad de Ingeniería Química, Universidad Nacional del Callao, Callao 09250, Peru

**Keywords:** Pitahaya, pitaya, *Hylocereus megalanthus*, drying kinetics models, Box–Behnken design, FTIR spectrum, texture profile analysis

## Abstract

The main objective of this study was to optimize the extraction of dietary fiber (insoluble dietary fiber and soluble dietary fiber) and degree of esterification from yellow dragon fruit peels using ultrasound-assisted extraction. Additionally, the study aimed to investigate the potential application of this fiber as a fat replacement in alpaca-based sausages. The optimization process for extracting dietary fiber and degree of esterification involved considering various factors, including the liquid-to-solid ratio, pause time, and total ultrasound application time. A Box–Behnken design consisting of 15 treatments was employed to determine the optimal levels for ultrasound-assisted extraction. The optimized conditions were found to be a liquid-to-solid ratio = 30 mL/g, pause time = 1 s, and total ultrasound application time = 60 min, which resulted in the highest values of insoluble dietary fiber (61.3%), soluble dietary fiber (10.8%), and the lowest value of degree of esterification (39.7%). The predicted values were validated against experimental data and showed no significant differences (*p* > 0.05). Furthermore, a completely randomized design was utilized to assess the effect of dietary fiber on replacing fat content during the production of alpaca-based sausages. The findings revealed that up to 78% of the fat content could be successfully replaced by soluble dietary fiber obtained from yellow dragon fruit peels when compared to high-fat sausages. Additionally, experimental sausages using soluble dietary fiber showed similar (*p* > 0.05) quality characteristics, such as hardness (24.2 N), chewiness (11.8 N), springiness (0.900), cohesiveness (0.543), redness (a* = 17.4), and chroma values (20.0), as low-fat commercial sausages.

## 1. Introduction

Fruit residues (peels, seeds, calyx, or pedicels) from the agri-food business comprise a considerable amount of organic matter, with the potential for processing and lowering environmental impacts [1]. Yellow dragon fruit (*Hylocereus megalanthus*) is widely employed in agriculture, resulting in more waste output than red dragon fruit [2]. The peel alone accounts for a significant portion (20–40%) of the fruit weight [3], making it essential for use as a byproduct in the food sector. It is rich in bioactive compounds, such as betaxanthins, betacyanins, ascorbic acid, and carotenoids [4,5] including dietary fiber (DF) as well. DF consists of several non-starch polysaccharides, such as cellulose, hemicellulose, pectin, and lignin. These components are resistant to enzymatic breakdown in the gastrointestinal tract, which regulates the intestinal transit and reduces cholesterol and blood glucose [6,7].

Enzymatic and chemical extraction procedures utilizing inorganic acids, alkalis, and organic acids are routinely used to extract DF from dragon fruit peels [8,9]. In this context, citric acid, a generally recognized as safe compound, is used in ultrasound-assisted extractions to increase yields by altering the structures of insoluble dietary fiber (IDF) and releasing cross-linked soluble dietary fiber (SDF) [9,10,11,12]. The application of ultrasonic waves, whose cavitation as a mechanism of action is caused by the rupture of bubbles [13], increases the shear capacity, causing a break of macromolecular structures [12]. In addition, Du et al. [9] mentioned that the application of ultrasound has increased the extraction yield of SDF. However, modifications of some properties of DF, such as molecular size and food texture, have been detected [9]. Additionally, the application of pulsed or intermittent ultrasound has been shown to decrease this damage of DF [14,15].

Statistical-mathematical tools, such as response surface methodology, may be used to improve generic extraction methods and enhance process development [16,17,18]. The Box–Behnken design (BBD), which integrates a fractional factorial design, allows for the optimization of extraction methods while lowering the number of experimental assays during the extraction of dietary fiber [19]. Furthermore, the BBD model employs central points, thereby mitigating experimental error [20]. Consequently, BBD methodology stands out as one of the most adaptable approaches for data analysis in experimental design, particularly due to its suitability to modify the levels of all factors using only three values (−1, 0, +1) that are evenly spaced [21]. Hence, it finds widespread application in the optimization of extraction parameters for dietary fiber from dragon fruit peel including pectin [1].

Low-degree esterified (DE) pectins, used in the formulation of low-fat emulsified sausages [22], have both health-promoting [23,24] and positive techno–functional qualities that improve the manufacturing process [25]. Fiber incorporation enables the manufacture of low-fat emulsified sausages from lean meats [22]. In this context, alpaca (*Vicugna pacos*), a lean meat source produced from South American camelids in the Andean highlands, might be used [26].

This research aims to (i) determine the optimal drying temperature and kinetics model for dragon fruit peels; (ii) optimize ultrasound-assisted extraction parameters, including liquid-to-solid ratio (LSR), pause time (PT), and total ultrasound application time (TUAT), for extracting DF (SDF and IDF) and DE from yellow dragon fruit peels; and (iii) investigate the utilization of IDF, SDF, and their mix (IDF/SDF = 1/1) as a fat replacement in alpaca-based sausages.

## 2. Materials and Methods

### 2.1. Plant Materials and Chemicals

The yellow ‘Palora’ dragon fruit (*Hylocereus megalanthus*) was provided by the Corporation Abregú SAC (Latitude: 11°25′00.7″ S; Longitude: 77°14′01.5″ W), Lima (Peru). Distilled water, citric acid (Sigma–Aldrich, Steinheim, Germany) and 96% ethanol (Hersil, Peru) were purchased from the local market.

### 2.2. Drying and Modeling of the Kinetics of Dragon Fruit Peels

#### 2.2.1. Drying of Dragon Fruit Peels

Fresh dragon fruits were washed and peeled (Figure S1). The peels were then cut into 3.5 × 0.5 cm segments and dried in a home-made tray dryer at 40, 55, and 70 °C at an air-flow rate of 0.5 m/s until a constant weight was reached. Using a data acquisition system comprised of a load cell coupled to an Arduino UNO, weight measurements were recorded over time. The particulate size was then reduced to 250 µm (W.S. Tyler, Mentor, OH, USA) using a cutting mill (RETSCH, SR300, Haan, Germany) to obtain dragon fruit peel powder (DFPP). Finally, the DFPP was stored in glass containers with hermetic seals.

#### 2.2.2. Modeling of Drying Kinetics

Using the models shown in Table S1, drying kinetics at 40, 55, and 70 °C were mathematically modeled. In addition, each model’s coefficients were determined by nonlinear fitting and evaluated using the coefficient of determination (R^2^), relative squared error (RSE), mean absolute percentage error (MAPE), and root mean square error (RMSE). Using Equation (1), the ratio of moisture (XR) was calculated:

(1) $$XR=\frac{M_{t}-X_{e}}{M_{0}-X_{e}}$$

where M_0_ is the initial moisture on a dry basis, M_t_ is the moisture on a dry basis at time ‘t’, and X_e_ is the equilibrium moisture that can be subtracted from Equation (1) due to the fluctuation of relative humidity during the drying period, according to Salehi et al. [27]. Consequently, XR was computed using Equation (2):

(2) $$XR=\frac{M_{t}}{M_{0}}$$

To determine the coefficients of the mathematical models for drying kinetics, non-linear regressions were used. The optimal model was chosen based on the parameters RMSE, RSE, R^2^, and MAPE.

#### 2.2.3. Effective Moisture Diffusivity (D_eff_)

As an unknown value, D_eff_ was calculated by fitting experimental data to Equation (3) derived from Fick’s second law [28] using the non-linear regression method:

(3) $$XR=\frac{8}{\pi^{2}}exp\left(-\frac{\pi^{2}\;D_{eff}\;t}{4L^{2}}\right)$$

where ‘L’ is half the thickness of the dragon fruit peel and ‘t’ is the drying time (hours).

### 2.3. Extraction, Yield of Dietary Fiber (DF), and Determination of Its Degree of Esterification (DE)

#### 2.3.1. Extraction of DF Fractions: SDF and IDF

A 1% (*w*/*v*) citric acid solution was prepared in distilled water. Each desiccated yellow dragon fruit peel (YDFP) sample obtained at 40, 55, and 70 °C was solubilized at a constant LSR concentration (30 mL/g) and heated at 90 °C for 2 h. IDF was identified as the solid fraction retained on the Whatman N°42 filter after filtration of the resulting solutions. Next, a 1:1 ratio (*v*/*v*) of 96% ethanol was added to the filtrate, resulting in the formation of SDF precipitate. Subsequently, both IDF and SDF were freeze-dried at −50 °C for 24 h, and the dehydrated products were stored in airtight glass containers for further analysis [10,11].

#### 2.3.2. Degree of Esterification (DE)

The DE values of SDF were determined using the titration method [29]. Initially, 0.2 g of SDF was placed in an Erlenmeyer flask and moistened with a few drops of 96% ethanol. Then, 20 mL of distilled water at 40 °C was added, and the mixture was stirred on a magnetic stirrer (Biosan, MSH-300, Madrid, Spain) for 2 h. The resulting solution was titrated with a 0.1 N NaOH solution (V_i_), with phenolphthalein serving as an indicator. Afterwards, 10 mL of the 0.1 N NaOH solution was added to the neutralized acid sample, and the solution was stirred for an additional 2 h. Subsequently, 10 mL of 0.1 N HCl was added. The excess HCl was then estimated using 0.1 N NaOH (V_f_). The DE value was calculated using Equation (4):

(4) $$DE\left(\%\right)=\frac{V_{i}}{V_{i}+V_{f}}\times 100\%$$

#### 2.3.3. Extraction Yield of DF Fractions (%)

The extraction yields of SDF and IDF were determined using Equations (5) and (6), where M_DFPP_ is the initial weight of DFPP, and M_IDF_ and M_SDF_ are the weights of IDF and SDF, respectively. Weights were measured with an electronic balance (OHASUS, PX523/E, China).

(5) $$\%{Yield}_{IDF}=\frac{M_{IDF}}{M_{DFPP}}\times 100$$

(6) $$\%{Yield}_{SDF}=\frac{M_{SDF}}{M_{DFPP}}\times 100$$

### 2.4. Ultrasound-Assisted Extraction and Characterization of Optimized Dietary Fiber (SDF and IDF)

#### 2.4.1. Optimization of Ultrasound-Assisted Extraction of IDF and SDF

Using an ultrasonic generator (Sonics & Materials, VC505, Connecticut, NN, USA) with a 12 mm ultrasound instrument diameter and a Box–Behnken experimental design (Table 1), the influence of three parameters was investigated. The generator’s output was 500 W, it operated at a frequency of 20 kHz, and its amplitude was 40%. The investigated variables included PT: 1, 3, and 5 s; LSR: 30, 50, and 70 mL/g; and TUAT: 15, 37.5, and 60 min. The response variables were the extraction of DF (SDF and IDF, %) and DE (%). A total of fifteen treatments were conducted.

Once the sample of dragon fruit peels powder suspended in 1% of citric acid (pH: 2, 100 mL) was treated with ultrasound at different conditions (Table 1), the resulting suspensions were filtered, and the solid fraction retained on the Whatman N°42 filter was IDF. Then, 96% ethanol was added to the filtrate in a 1:1 ratio (*v*/*v*), leading to the formation of a precipitate (SDF). Both IDF and SDF were subsequently freeze-dried at −50 °C for 24 h, and the dehydrated products were stored in sealed glass containers for further analysis. A total of fifteen treatments (Table 1), and the response variables were DF extraction (SDF, and IDF, %), and DE (%).

#### 2.4.2. Water-Holding Capacity (WHC) and Oil-Holding Capacity (OHC)

The WHC was determined using the method outlined by Valencia and Román [30]. Briefly, 0.5 g of each sample (P_0_) was weighed into 15 mL falcon tubes, followed by the addition of 10 mL distilled water. The mixture was manually stirred for ten minutes and then left at room temperature for 24 h. The samples were then centrifuged at 3000 rpm for 10 min. The supernatant was removed with caution, and the sediment’s (P_1_) mass in grams was recorded. Using Equation (7), WHC values were reported as grams of water per gram of sample:

(7) $$WHC=\frac{\left(P_{1}-P_{0}\right)}{P_{0}}\;$$

The OHC was computed using the methodology outlined by Chau and Huang [31]. In total, 1 g of IDF or SDF pulverized sample (P_0_) was added to 5 mL of soybean oil. The mixture was agitated for 30 s every 5 min for 30 min, and then centrifuged at 1600× *g* for 25 min. The remaining oil phase was eliminated, and the weight of the absorbed oil (O_a_) was calculated based on the difference between the initial sample’s weight and the final sample’s weight. The OHC was computed using Equation (8) and expressed as grams of retained oil per gram of fiber:

(8) $$OHC=\frac{O_{a}-P_{0}}{P_{0}}\;$$

#### 2.4.3. Swelling Capacity (SC)

The SC was determined using the technique outlined by Valencia and Román [30]. A total of 2 g of the sample (P) was deposited in a graduated cylinder containing 25 mL. After noting the initial volume (V_0_, mL), 10 mL of distilled water was introduced. The mixture was hand-shaken for 5 min and left at room temperature for 24 h. After that, the final sample volume (V_1_, mL) was recorded. Using Equation (9), the SC (mL/g) was computed:

(9) $$SC=\frac{V_{1}-V_{0}}{P}$$

#### 2.4.4. FTIR Analysis

Using an FTIR spectrophotometer (Perkin Elmer, Spectrum Two, Waltham, MA, USA), the functional groups of DFPP, SDF, IDF, and commercial pectin (CP) were determined. The ATR accessory was used to record FTIR spectra in the range of 4000–400 cm^−1^ with a resolution of 4 cm^−1^ and 32 scans [32].

### 2.5. Application of Dietary Fibers Fractions in Alpaca-Based Sausage

The extracted fibers (IDF and SDF) and their mix (IDF/SDF = 1/1) were incorporated into emulsified alpaca-based sausages in proportions determined by trial-and-error experiments. The qualitative characteristics of the sausages were then evaluated.

#### 2.5.1. Elaboration of Low-Fat Frankfurter-Type Sausages

Pieces of frozen (−20 °C) alpaca (*Vicugna pacos*) meat *(Longissimus dorsi* muscle from a 24-month-old male alpaca) were pulverized in a grinder machine (Torrey, M-32-3HP, Mexico City, Mexico), and the ingredients listed in Table 2 were added in the following order: (1) alpaca meat, sodium chloride, nitrite, sugar, erythorbate, and sodium polyphosphate made up the first ingredients. These ingredients were mixed for one min and left to rest for 3 min; (2) one-third of cold water at 1 °C was added and mixed for one minute; (3) soy protein concentrate, skim milk powder, and a third of cold water were added and mixed until a homogeneous mass was obtained; (4) pork backfat was incorporated only for Control 1 (C1); and then, (5) potato starch was added along with the remaining cold water, and only in Control 2 (C2) was carrageenan added. A mixture of black pepper, cumin, and nutmeg granules, along with carmine colorant and smoke essence, was subsequently added. Using a sausage stuffer, the formed beef sausage bulk was packed into synthetic casings of 20 mm diameter. For further analysis, the sausages were cooked at 70 °C for 12 min and then chilled to 4 °C.

#### 2.5.2. Texture Profile Analysis (TPA)

Texture profile analysis was performed on sausage segments (18 mm in diameter and 15 mm in thickness) according to the modified technique described by Zhao et al. [33] using a Texture Analyzer (Brookfield Ametek, CTX, Middleborough, MA, USA) with a 5 kg load cell and a 38.1 mm cylindrical aluminum probe. Analyses were conducted with trigger, deformation, and test speed values of 0.05 N, 50%, and 2 mm/s, respectively.

#### 2.5.3. Cooking Losses (CL), Water Activity (a_w_), and Processing Yield (PY)

CL was determined using the procedure outlined by Wongpattananukul et al. [34]. Before (W_1_) and after (W_2_) cooking, the sausages were weighed, and Equation (10) was applied:

(10) $$CL=\frac{W_{1}-W_{2}}{W_{1}}\times 100\%$$

where a_w_ is measured in duplicate at 25 °C using 10 g of minced sausage and AquaLab apparatus (4TE Decagon, Meter Group, Pullman, WA, USA) [35]. PY was determined by subtracting the weight of the uncooked sausage from the weight of the cooked sausage. The results were reported as a percentage (%).

#### 2.5.4. Instrumental Color Analysis

Using a colorimeter (KONICA MINOLTA, CR-400, Tokyo, Japan), color measurements were taken in the CIE L* a* b* color space in accordance with the procedure described by Oshima et al. [36]. The chroma values and sexagesimal degrees of hue° were calculated as indicators of color saturation and the red/yellow ratio using Equations (11) and (12), respectively:

(11) $$Chroma=\sqrt{{a^{*}}^{2}+{b^{*}}^{2}}$$

(12) $$Hue{}^{\circ}=\frac{180}{\pi }\times arctan\left(\frac{b^{*}}{a^{*}}\right)$$

#### 2.5.5. Chemical Composition of the Alpaca-Based Sausage including Dietary Fiber from DFPP

The levels of moisture (930.15), crude protein (920.152), fat (930.09), ash (920.108), crude fiber (978.10), iron (975.03), and zinc (975.03) were determined using official AOAC procedures (AOAC, 2019) [37]. The carbohydrate content of the substance can be calculated using the following formula: %carbohydrates = 100 − (%moisture + %crude protein + %fat + %ash).

### 2.6. Statistical Analysis

All the results are presented as the mean ± standard deviation (SD) of at least three independent, triplicate measurements. A one-way analysis of variance (ANOVA) was conducted with a significance level of 5% (α = 0.05) to ascertain statistical differences between the fifteen treatments and six formulations of alpaca-based sausages. When the ANOVA was significant (*p* < 0.05), the LSD–Fisher test was used. Using Design Expert (version 9.0.6.2, Stat-Ease Inc., Minneapolis, MN, USA), a Box–Behnken design was used to optimize the extraction of the IDF, SDF, and DE, obtaining a three-dimensional (3D) response surface plot, an ANOVA and the fitting quality (lack of fit and adjusted R^2^) parameters. The applied polynomial model was Equation (13):

(13) $$Y=\beta_{0}+\sum_{i=1}^{k}\beta_{i}x_{i}+\sum_{i=1}^{k}\beta_{ii}x_{i}^{2}+\sum_{i=1}^{k}\sum_{j=1}^{k}\beta_{ij}x_{i}x_{j}+\varepsilon$$

In the Equation (13), Y is the dependent variable, β_0_ is the independent term, β_i_ are the linear regression coefficients, β_ii_ are the quadratic regression coefficients, β_ij_ are the interaction regression coefficients, x_i_ or x_j_ and the independent variables or factors, and k is the number of independent variables or factors. To compare the experimental vs. theoretical values, a validation analysis was performed at a 95% confidence level, where a *p*-value greater than 0.05 means no significant statistical difference.

The RStudio software (R Foundation for Statistical Computing, Vienna, Austria) was employed for modeling drying curves, estimating fitting parameters, and performing one-way ANOVA. The following packages were used: ‘gslnls’ library version 1.1.1 [38], ‘Metrics’ library version 0.1.4 [39], ‘stats’ version 4.2.2, and ‘agricolae’ version 1.3–5 [40].

## 3. Results and Discussion

### 3.1. Study of the Drying Kinetics and Effect of Drying Temperature on Dragon Fruit Peels

**Figure 1:**
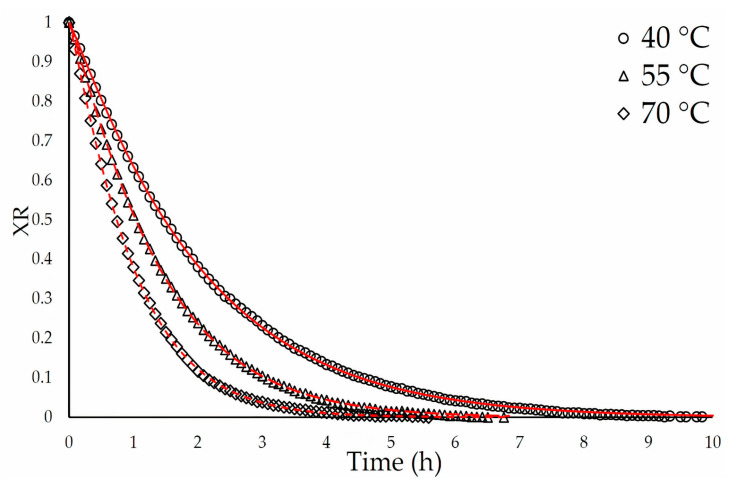
Using experimental data fitted by Page’s model (red line) to the drying curves of dragon fruit peels at various temperatures: 40 °C (○), 55 °C (△), and 70 °C (◊). XR: moisture ratio vs. time (hours).

Eleven mathematical models (Table S1) were assessed to determine the most accurate drying kinetics model. Based on the highest R^2^ value (0.9999) and the lowest RMSE (3.59), RSE (1.97) and MAPE (2.0) values (Table S2), the Page model was selected as the best fit. This equation has been demonstrated to be a useful method for modeling the drying kinetics of a variety of fruits and vegetables [1,41]. In addition, Hameed et al. [42] and Liemohn et al. [43] suggested that Page’s model was chosen due to its superior accuracy and predictive ability compared to other models. The drying temperatures applied to YDFP had a notable influence on XR and D_eff_ (Table 3), where higher temperatures resulted in shortened water loss times (Figure 1). This effect was most evident in the variation of the coefficient (k_0_) of Page’s model (Table S3). In accordance with the findings of Kang et al. [44], who observed a rise in D_eff_ at higher drying temperatures in algae, this indicates a rapid removal of water as the temperature rises. Mewa et al. [28] and Ali et al. [45] both reported comparable findings.

The color analysis revealed that lower drying temperatures (40 °C) darkened dragon fruit peels, as indicated by decreased luminosity (L*) and increased redness (a*) values. In contrast, higher drying temperatures (70 °C) and shorter drying times (5.25 h) led to greater L* values for DFPP, indicating a lower concentration of dark melanoid compounds produced by the Maillard reaction. At 50 and 70 °C, the L* values were statistically comparable (*p* > 0.05), whereas at 40 °C, a mild discoloration was observed. The a* values were greater (*p* < 0.05) at 40 °C, indicating the preservation of red pigmentation at lower temperatures, whereas pigment degradation was greater at 55 and 70 °C (*p* > 0.05), as previously reported by Thewes et al. [46] and Reis et al. [47] in pecans drying. At 70 °C, the yellowness parameter (b*) indicated greater carotenoid retention, whereas at 55 °C, partial pigment degradation occurred, which could negatively affect the color of the sausages. The drying treatment at 55 °C produced the maximum quantities of IDF and SDF (Table 3), which is consistent with the findings of Chua et al. [1], who reported similar results at 45 °C. Given our objective of obtaining a high DF content, 55 °C was the optimal working temperature.

### 3.2. Optimization of Dietary Fiber Extraction Parameters from DFPP

Treatment T_2_ demonstrated the highest yield (61.4%) of IDF extraction, which was statistically similar (*p* > 0.05) to treatments T_1_, T_7_, T_9_, and T_11_, as shown in Table 4. Similarly, treatment T_3_ achieved the highest yield (12.0%) of SDF extraction, with PT = 1 s, LSR = 70 mL/g and TUAT = 37.5 min. The response variable DE exhibited significant (*p* < 0.05) differences among the treatments, with the maximum value observed in treatment T_6_ (58.1%). However, from a techno–functional perspective, a lower DE is more favorable as it promotes a higher gelling power and water-holding capacity of pectin-rich dietary fiber when exposed to calcium ions [48], as demonstrated by treatment T_1_ (41.1%), which was statistically comparable (*p* > 0.05) to treatments T_3_, T_7_, and T_11_.

**Figure 2:**
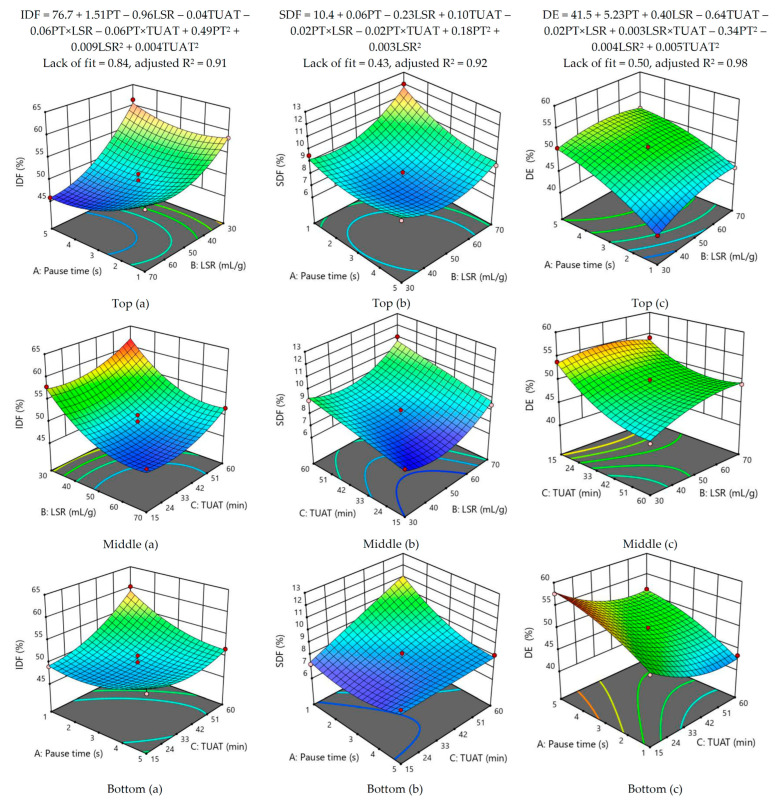
Insoluble dietary fiber (IDF, **a**), soluble dietary fiber (SDF, **b**), and degree of esterification (DE, **c**) responses surface in 3D. **Top**: pause time (A) vs. liquid-to-solid ratio (B). Liquid-to-solid ratio (LSR, B) vs. total ultrasonic application time (TUAT, C) in the middle. **Bottom**: Total ultrasonic application time (TUAT, C) vs. pause time (PT, A).

The significance of the mathematical models for IDF, SDF, and DE was evaluated by calculating *p*-values, and only those with a significant level (*p* < 0.05) were considered. Non-significant terms were then eliminated from the model, resulting in the development of a reduced quadratic model. This new model showed a substantial increase in statistical significance, reaching a highly significant level (*p* < 0.001) as indicated in Table S4. Specifically, the terms LSR×TUAT, LSR×TUAT, TUAT^2^, and PT×TUAT were removed for IDF, SDF, and DE, respectively. The lack of fit demonstrated non-significance (*p* > 0.05), indicating a good fit of each model to the data. The lowest adjusted R^2^ value was observed in the response surface model for IDF (0.90). However, after performing the reduction of non-significant terms, the adjusted R^2^ value increased to 0.91, and the value of the sum of squares of the predicted residuals (PRESS) was reduced (Table S4). According to Encina–Zelada et al. [35], a higher PRESS value indicates lower predictive quality, so it is recommended to eliminate non-significant terms to reduce PRESS and increase R^2^. The adjusted R^2^ values for all response surface models were above 0.90 (Figure 2), indicating the suitability of these models. Additionally, the equations of the predictive models are shown in Figure 2, being all adjusted R^2^ values greater than 0.90 and the values of the lack of fit test were non-significant (*p* > 0.05), indicating good fit to experimental data [49].

The extraction of IDF was optimized by using the following parameters: PT = 5 s and LSR = 30 mL/g (Figure 2, top-a); TUAT = 60 min and LSR = 30 mL/g (Figure 2, middle-a); and TUAT = 60 min and PT = 1 s (Figure 2, bottom-a). In the case of SDF, the best extraction was achieved by using LSR = 70 mL/g and PT = 1 s (Figure 2, top-b); LSR = 70 mL/g and TUAT = 60 min (Figure 2, middle-b); and TUAT = 60 min and PT = 1 s Figure 2, bottom-b). The lowest DE value was achieved by using PT = 1 s and LSR = 30 mL/g (Figure 2, top-c), TUAT = 60 min and LSR = 30 mL/g (Figure 2, middle-c) and TUAT = 60 min and PT = 1 s (Figure 2, bottom-c). As a result, the optimization criteria for IDF yield was maximized by giving it a significance score of three (+++). SDF, a critical component with a variety of health advantages [23,24] and prospective uses in the food industry [50], notably in the formulation of leaner meat products [22,51,52,53,54], was optimized by giving it a high significance score of five (+++++) to produce the highest SDF production. The optimization criteria for DE were minimized with a three (+++) significance score in order to apply it to meat products such as sausages, where there is neither a high sugar content nor a pH lower than three. According to Thibault and Rinaudo [48], in the presence of calcium ions, it is feasible to get gel formation with strong gelling ability at a neutral pH by working with a pectin containing less than 40% DE.

The best extraction conditions were obtained by maximizing variables IDF and SDF and reducing DE at the following parameters: PT = 1 s, LSR = 30 mL/g, and TUAT = 60 min. Statistically equivalent values (*p* > 0.05) were found during the validation step (working at a 95% confidence level) between the expected (theoretical) and experimental yields (IDF, SDF, and DE) (Table 5).

### 3.3. Characterization of Optimized Dietary Fiber

#### 3.3.1. Techno–Functional Properties (WHC, OHC, SC) of Dietary Fibers

WHC determination was not achievable because of SDF’s high solubility in water, resulting in a clear solution devoid of insoluble particles (Table 6). Other research using the same methodology has shown high WHC values for SDF, such as 5.26 g/g for papaya peels [49] and 13.4 g/g for grapefruit peels [55]. IDF, on the one hand, had a WHC of 11%, which might be attributed to its ability to retain water and swell within the porous matrix. SDF, on the other hand, exhibited different water-interacting properties, such as gelation in the presence of calcium ions. Fiber concentrate has a lower WHC value of 4.1 g/g [30], but IDF from bean hulls has a WHC of 4.66 g/g [56]. IDF is acceptable for application in food formulations because of the high WHC values obtained [55].

Due to the lack of lipophilic functional groups that may interact with non-polar molecules such as oil, SDF has low OHC values (Table 6). However, our findings exceeded the values published by Zhang et al. [49] (1.40 g/g), which might be related to changes in the extraction pH altering SDF characteristics. Xie et al. [55], on the one hand, produced an OHC of 22.1 g/g employing a microwave treatment at 85 °C, demonstrating the effect of extraction procedures and raw materials on SDF characteristics linked to OHC. IDF, on the other hand, had a substantial OHC value of 5.0 g/g (Table 6), owing to its fibrous and porous structure, which shows hydrophobic or non-polar functional groups, such as C−H stretching (−CH_3_ or =CH_2_) and ethers (C−O), capable of interacting with non-polar substances such as oil [57,58]. High OHC in IDF may help to inhibit dietary fat absorption and lower blood cholesterol and triglyceride levels, providing potential health advantages [23,55,56].

IDF had a higher SC (4.86 mL/g) (Table 6) than that reported by Yin et al. [56] (2.8 mL/g). This increase is most likely due to the cellulose components, which have been linked to SC [59]. As a result, IDF seems to be a potential option for increasing yield and lowering production costs in food formulations [55]. Furthermore, the increased SC of IDF, owing to its cellulose content, leads to enhanced intestinal transit [31].

#### 3.3.2. FTIR Analysis

**Figure 3:**
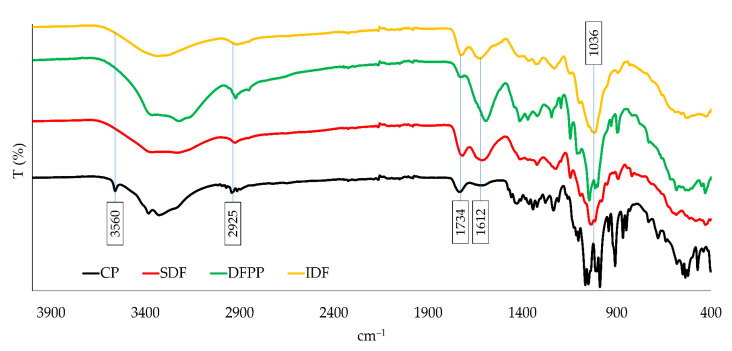
Commercial pectin (CP), soluble dietary fiber (SDF), dragon fruit peel powder (DFPP) and insoluble dietary fiber (IDF) FTIR spectra. T (%): percentage of transmittance vs. cm^–1^.

Five distinct peaks were found in the FTIR examination at the following wavelengths: 1036, 1612, 1734, 2925, and 3560 cm^−1^ (Figure 3). Carbohydrates are distinguished by the peculiar peak form seen at 1036 cm^−1^. According to Spinei and Oroian [32], the major chemical groups of the various polysaccharides may be distinguished between 1410 and 800 cm^−1^. Oliveira et al. [60] and Chua et al. [1], reported that non-esterified carboxylic groups are seen at wavelengths close to 1610 cm^−1^, a finding that is comparable to the peak seen in our study (1612 cm^−1^). The above-mentioned authors also noted that the peak at 1734 cm^−1^, which corresponds to the same wavelength as in Figure 3, is due to the stretching vibration of the methyl-esterified carboxylic groups. In comparison to CP, SDF, and IDF, DFPP showed the lowest peak at 1734 cm^−1^. Since DFPP is a raw sample, this disparity may be attributed to the lack of extracted dietary fiber (IDF or SDF). The peak at 3415 cm^−1^ corresponds to hydroxyl groups (−OH), whereas the peak at 2936 cm^−1^ belongs to various methyl groups (−CH, −CH_2_, −CH_3_) [60]. As the SDF had more non-esterified carboxylic groups (1612 cm^−1^), as opposed to methyl-esterified carboxylic groups (1734 cm^−1^), the DE should have been lower [61]. The acid–base titration technique, which indicates a DE of 39.7%, may classify SDF as low methyl-esterified pectin (<50% DE) as reported by Reichembach and Oliveira [62] (Table 5), who supported this assumption.

### 3.4. Effect of the Application of Dietary Fiber in the Production of Alpaca-Based Sausages

Four formulations with various types of dragon fruit fiber (DFPP, IDF, SDF, and an IDF/SDF mix) were compared to two controls. F1, F2, F3, and F4 represented DFPP, IDF, SDF, and a mix of IDF/SDF (1/1), respectively. The controls were a high-fat formulation without dragon fruit fiber (Control 1, C1) and a low-fat formulation including carrageenan (Control 2, C2).

#### 3.4.1. Texture Profile Analysis (TPA)

The formulation of F3 sausage, which contained SDF, was comparable (*p* > 0.05) to that of C2, which contained carrageenan and was also a low-fat commercial formulation (Table 7). The increased SDF content in F3 led to greater (*p* < 0.05) hardness compared to the other formulations (F1, F2, or F4), contributing to increased gel strength through the construction of three-dimensional networks driven by pectin and calcium ion interactions [48]. All low-fat formulations, including C2, demonstrated lower hardness (*p* < 0.05) than C1. It is possible that the quantity of water contained in their formulations (Table 2) led to the reduction in sausage hardness. Therefore, it can be said that the firmness of low-fat sausages is typically lower than that of traditional formulations [25,63]. In contrast, the chewiness value of formulation C2 was comparable (*p* > 0.05) to that of formulation F3 (Table 7), confirming that this formulation has a similar hardness to commercially available low-fat sausage, as reported by [63] who obtained similar hardness values (~21.7 N). The F3 formulation had a similar (*p* > 0.05) textural profile consisting of hardness, chewiness, springiness, adhesiveness, and shear stress to C2.

#### 3.4.2. Evaluation of Color Parameters of Frankfurter-Type Sausages and Low-Fat Alpaca-based Sausages

**Figure 4:**
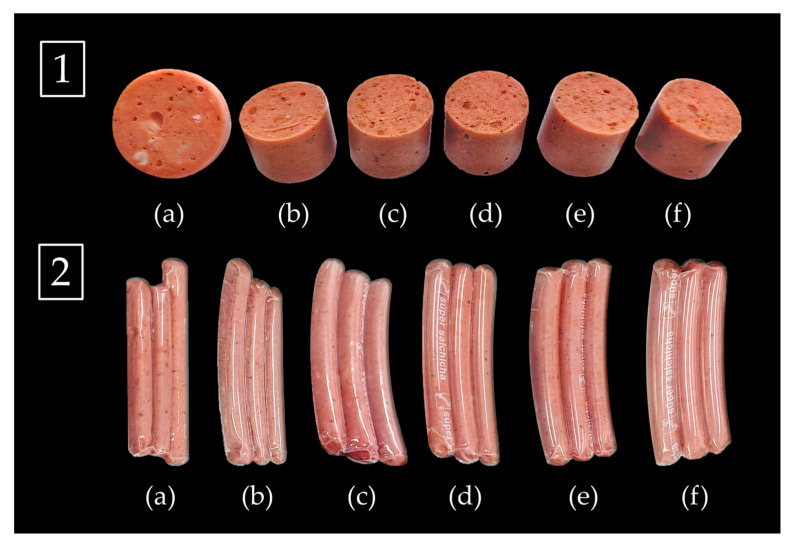
Images of alpaca-based sausages, in cross-section (**1**) and in their entirety (**2**). C1: formulation with high-fat content (**a**), C2: formulation with low-fat carrageenan (**b**), F1: formulation with dragon fruit peel powder (DFPP) (**c**), F2: formulation with insoluble dietary fiber (IDF) (**d**), F3: formulation with soluble dietary fiber (SDF) (**e**), and F4: formulation with IDF/SDF = 1/1 (**f**).

In comparison to C1, formulations F1, F2, F3, and F4 showed enhanced visual macrostructural appearance without white marbled fat (Figure 4(1c–f)). The statistically equivalent (*p* > 0.05) greater L* values in formulations F1 (DFPP), F3 (SDF), and F4 (mix of IDF/SDF) indicated improved lightness. C1, in comparison, had the lowest L* value. In comparison to C2, formulations F1 (DFPP), F2 (IDF), and F3 (SDF) had higher chroma values, which indicated a more vivid red color (a*). In comparison to C2, all fiber-containing sausages (F1–F4) had considerably (*p* < 0.05) higher b* values. These results diverge from those published by Abbasi et al. [63], who found that the L*, a*, and b* values of low-fat sausages using gum tragacanth showed no statistically significant changes. Consumers prefer products with high L* values and low a* values, according to Nollet and Toldra [64], since a reddish tint may be a sign that artificial colorants were used.

The hue° values range from 0 to 90°, where the greater number denotes increasing yellow color intensity [46]. The chroma value expresses the color purity. Therefore, the IDF, SDF, and DFPP samples’ chroma levels suggested greater color purity. The fact that all samples had low hue° values (*p* < 0.05) further suggests that red is the predominant color in the study’s sausage samples. These color parameters may be compared to the 19.0 and 22.5° values in chroma and hue°, respectively, achieved from veal sausages [65].

#### 3.4.3. Evaluation of Cooking Losses (CL), Water Activity (a_w_), and Processing Yield (PY)

The water activities of F3 and F4 were comparable (*p* > 0.05), but greater than C1 (Table 7). This is likely due to the fact that the SDF present in formulations F1, F2, F3, and F4 retains more water (Table 2). F1 and F3 also had a higher PY, but the difference was not statistically significant (*p* > 0.05). Then, a higher value for CL was obtained for formulation C1 (with a high-fat content), most likely because this formulation contained less water, indicating that the lower the water activity, the smaller the cooking losses (Table 7). In contrast, another study found that adding orange fibers (1–4%) to beef sausages without reducing the lipid content had no effect on water activity [54].

#### 3.4.4. Chemical Analysis in an Alpaca-Based Sausage including SDF Obtained from DFPP

Abbsi et al. [63], who attained 7.16% of fat in beef-based sausages, reported a much higher fat content in their beef-based sausages than the F3 sausage (4.01%, Table 8). It should be noted that alpaca meat has a lower intramuscular fat content than beef, which contributes to the lower fat content in sausages [65,66]. In contrast, Yadegari et al. [53] reported 10.82% fat in beef-based sausages with 1% fat reduction derived from methyl cellulose. In addition, alpaca meat contains important minerals, such as iron and zinc, necessary for a healthy and balanced diet that contributes to strong brain development, immune system function, and reduction in nutritional disorders including anemia [66].

In the framework of a future research perspective, further exploration should be performed to determine the structural composition of DFPP dietary fibers, including the polysaccharide structure utilizing advanced techniques such as two-dimensional nuclear magnetic resonance spectroscopy (2D NMR). Additionally, the potential application of these fiber-rich ingredients, obtained from DFPP, as fat substitutes in different meat products (i.e., hamburgers, nuggets, hot dogs) is being undertaken in the laboratories of our group and will be evaluated. Comprehensive sensory evaluations will subsequently be conducted to assess the acceptability of these low-fat meat products.

## 4. Conclusions

Yellow dragon fruit peels may be utilized as a source of soluble and insoluble dietary fiber, and their fiber can be used to substitute fat in the production of sausages with alpaca meat. The best dietary fiber level was obtained by drying yellow dragon fruit peels at 55 °C, with the Page mathematical model being the most suitable equation to model the drying process at this temperature.

According to the findings of the optimization approach using statistical surface response methodology, via the Box–Behnken design, the optimized values for insoluble dietary fiber, soluble dietary fiber, and degree of esterification were 61.3%, 10.8%, and 39.7%, respectively, values that were properly validated (*p* > 0.05). 

Last but not least, the incorporation of soluble dietary fiber in the alpaca-based sausages revealed parameters that were comparable (*p* > 0.05) to those of a low-fat commercial formulation, including hardness, chewiness, springiness, cohesiveness, redness, and chroma. Furthermore, it exhibited a 78% reduction in fat content when compared to the high-fat commercial formulation. These findings suggest that incorporating novel additives, such as soluble dietary fiber derived from yellow dragon fruit peels, using an environmentally friendly technique such as ultrasound-assisted extraction, is promising to develop sausages with a reduced fat content.

## Figures and Tables

**Table 1:** Box–Behnken experimental design applied for ultrasound-assisted extraction of total dietary fiber, soluble (SDF) and insoluble (IDF), and degree of esterification (DE).

Treatment	X_1_	X_2_	X_3_
T_1_	1.00 (–1)	30.0 (–1)	37.5 (0)
T_2_	5.00 (1)	30.0 (–1)	37.5 (0)
T_3_	1.00 (–1)	70.0 (1)	37.5 (0)
T_4_	5.00 (1)	70.0 (1)	37.5 (0)
T_5_	1.00 (–1)	50.0 (0)	15.0 (–1)
T_6_	5.00 (1)	50.0 (0)	15.0 (–1)
T_7_	1.00 (–1)	50.0 (0)	60.0 (1)
T_8_	5.00 (1)	50.0 (0)	60.0 (1)
T_9_	3.00 (0)	30.0 (–1)	15.0 (–1)
T_10_	3.00 (0)	70.0 (1)	15.0 (–1)
T_11_	3.00 (0)	30.0 (–1)	60.0 (1)
T_12_	3.00 (0)	70.0 (1)	60.0 (1)
T_13_	3.00 (0)	50.0 (0)	37.5 (0)
T_14_	3.00 (0)	50.0 (0)	37.5 (0)
T_15_	3.00 (0)	50.0 (0)	37.5 (0)

The values in parentheses correspond to the coded values. Studied factors: X_1_: Pause time (PT, s), X_2_: Liquid-to-solid ratio (LSR, mL/g), X_3_: Total ultrasound application time (TUAT, min).

**Table 2:** Different sausage formulations (%) with addition of dragon fruit peel powder (DFPP), insoluble dietary fiber (IDF), soluble dietary fiber (SDF), and its IDF/SDF mix.

Ingredient (%)	C1	C2	F1	F2	F3	F4
Alpaca meat	48.96	48.96	48.96	48.96	48.96	48.96
Pork back fat	23.10	-	-	-	-	-
Water	16.65	33.95	33.87	33.95	33.76	33.52
Oil	-	5.10	5.10	5.10	5.10	5.10
SDF	-	-	-	-	0.80	0.54
IDF	-	-	-	0.70	-	0.54
Calcium chloride	-	-	0.08	-	0.08	0.06
DFPP	-	-	0.70	-	-	-
Carrageenan	-	0.70	-	-	-	-
Sodium chloride	1.73	1.73	1.73	1.73	1.73	1.73
Curing salt (20% nitrites)	0.04	0.04	0.04	0.04	0.04	0.04
Sodium erythorbate	0.07	0.07	0.07	0.07	0.07	0.07
Sodium polyphosphate	0.39	0.39	0.39	0.39	0.39	0.39
Sugar	0.39	0.39	0.39	0.39	0.39	0.39
Potato starch	3.92	3.92	3.92	3.92	3.92	3.92
Soy concentrate	1.96	1.96	1.96	1.96	1.96	1.96
Milk powder	2.15	2.15	2.15	2.15	2.15	2.15
Black pepper	0.15	0.15	0.15	0.15	0.15	0.15
Cumin powder	0.15	0.15	0.15	0.15	0.15	0.15
Nutmeg	0.15	0.15	0.15	0.15	0.15	0.15
Monosodium glutamate	0.05	0.05	0.05	0.05	0.05	0.05
Carmine colorant	0.10	0.10	0.10	0.10	0.10	0.10
Smoke essence	0.04	0.04	0.04	0.04	0.04	0.04

C1: high-fat Frankfurter-type sausage formulation, C2: low-fat Frankfurter-type sausage with carrageenan formulation, F1: dragon fruit peel powder formulation, F2: insoluble dietary fiber formulation, F3: soluble dietary fiber formulation, and F4: mix of IDF and SDF (1/1) formulation.

**Table 3:** The drying time, effective moisture diffusivity (D_eff_), color parameters (L*, a*, and b*), and yields of dietary fiber (DF) obtained from dragon fruit peel powder (DFPP) dried at different temperatures.

Analysis		Temperature (°C)		
		40	55	70
Drying	Time (h)	10.0 ± 0.3278 ^a^	6.24 ± 0.0610 ^b^	5.25 ± 0.4637 ^c^
	D_eff_ (m^2^/s)	1.02 × 10^–10^ ± 3.75 × 10^–12 c^	1.49 × 10^–10^ ± 1.06 × 10^–11 b^	2.10 × 10^–10^ ± 2.15 × 10^–11 a^
Color	L*	72.0 ± 1.172 ^b^	74.6 ± 0.973 ^a^	76.2 ± 0.057 ^a^
	a*	7.04 ± 0.290 ^a^	3.79 ± 0.497 ^b^	4.22 ± 0.735 ^b^
	b*	27.1 ± 0.373 ^b^	27.9 ± 0.800 ^b^	31.4 ± 0.318 ^a^
Yield (%)	IDF	51.2 ± 0.220 ^b^	51.8 ± 0.337 ^a^	51.7 ± 0.122 ^ab^
	SDF	8.60 ± 0.211 ^a^	8.34 ± 0.070 ^a^	7.49 ± 0.078 ^b^

Values are presented as means ± SD (*n* = 3). D_eff_: Effective diffusivity. Different letters within the same row indicate statistically significant differences (*p* < 0.05).

**Table 4:** Yields for insoluble dietary fiber (IDF) and soluble dietary fiber (SDF), as well as determination of degree of esterification (DE).

Treatment	IDF (%)	SDF (%)	DE (%)
T_1_	58.5 ± 0.10 ^a^	9.54 ± 0.27 ^c^	41.1 ± 1.56 ^f^
T_2_	61.4 ± 1.60 ^a^	8.77 ± 0.05 ^def^	49.5 ± 1.50 ^de^
T_3_	51.8 ± 2.14 ^b^	12.0 ± 0.21 ^a^	43.1 ± 1.33 ^f^
T_4_	45.8 ± 1.71 ^c^	8.82 ± 0.63 ^de^	51.7 ± 0.73 ^cd^
T_5_	49.0 ± 1.75 ^bc^	7.22 ± 0.45 ^j^	48.1 ± 1.24 ^e^
T_6_	51.2 ± 1.51 ^b^	8.13 ± 0.07 ^fghi^	58.1 ± 0.76 ^a^
T_7_	60.2 ± 0.38 ^a^	10.8 ± 0.08 ^b^	41.7 ± 2.48 ^f^
T_8_	51.6 ± 0.91 ^b^	8.09 ± 0.31 ^ghi^	50.5 ± 1.26 ^cde^
T_9_	57.9 ± 2.09 ^a^	7.60 ± 0.46 ^hij^	55.4 ± 2.31 ^ab^
T_10_	47.8 ± 0.11 ^bc^	8.45 ± 0.22 ^efg^	52.7 ± 2.04 ^bc^
T_11_	60.8 ± 1.29 ^a^	9.13 ± 0.09 ^cd^	43.8± 0.03 ^f^
T_12_	51.7 ± 0.93 ^b^	11.0 ± 0.11 ^b^	50.0 ± 1.46 ^cde^
T_13_	46.7 ± 2.95 ^c^	7.82 ± 0.30 ^ghij^	49.8 ± 0.37 ^cde^
T_14_	50.8 ± 4.16 ^b^	7.51 ± 0.39 ^ij^	49.5 ± 0.78 ^de^
T_15_	49.3 ± 2.04 ^bc^	8.19 ± 0.13 ^efgh^	49.4 ± 0.49 ^d^

Values represent the means ± SD (*n* = 3). Different letters in the same column indicate statistical differences (*p* < 0.05).

**Table 5:** Comparison of extraction yields for insoluble dietary fiber (IDF) and soluble dietary fiber (SDF), as well as degree of esterification (DE), at 95% confidence interval (lower and upper limits).

Dependent Variables	Lower Limit	Upper Limit	Predicted Value	Experimental Value
IDF (%)	61.1	70.1	65.6 ^a^	61.3 ± 0.578 ^a^
SDF (%)	9.98	12.2	11.1 ^b^	10.8 ± 0.162 ^b^
DE (%)	36.0	39.7	37.8 ^c^	39.7 ± 0.374 ^c^

Values represent the means ± SD (*n* = 3). Similar letters in the same row indicate no significant differences (*p* > 0.05).

**Table 6:** Techno–functional characteristics (WHC: water-holding capacity; OHC: oil-holding capacity; SC: swelling capacity) of the dietary fiber by optimal parameters (IDF: insoluble dietary fiber; SDF: soluble dietary fiber).

Dietary Fiber	WHC (g/g)	OHC (g/g)	SC (mL/g)
IDF	11.0 ± 0.240	5.00 ± 0.347 ^a^	4.86 ± 0.031
SDF	n.d.	0.33 ± 0.011 ^b^	n.d.

Values represent the means ± SD (*n* = 3), n.d.: not detected. Different letters in the same column indicate statistical differences (*p* < 0.05).

**Table 7:** Texture profile analysis (hardness, chewiness, springiness, cohesiveness, adhesiveness, rupture force, and shear stress), water activity (a_w_), processing yield (PY, %), cooking losses (%), and colorimetric analysis (L*, a*, b*, chroma, and hue°) of alpaca-based sausages produced by different formulations (C1, C2, F1, F2, F3, and F4).

Analysis	C1	C2	F1	F2	F3	F4
Hardness (N)	43.6 ± 4.21 ^a^	22.6 ± 1.71 ^b^	16.2 ± 1.40 ^c^	12.8 ± 1.09 ^d^	24.2 ± 1.48 ^b^	17.3 ± 0.926 ^c^
Chewiness (N)	19.8 ± 1.36 ^a^	12.1 ± 1.28 ^b^	6.02 ± 0.768 ^c^	4.38 ± 0.554 ^d^	11.8 ± 1.37 ^b^	6.77 ± 0.683 ^c^
Springiness	0.800 ± 0.0001 ^d^	0.886 ± 0.0378 ^ab^	0.871 ± 0.0488 ^ab^	0.829 ± 0.0488 ^cd^	0.900 ± 0.0001 ^a^	0.857 ± 0.0535 ^bc^
Cohesiveness	0.571 ± 0.0488 ^ab^	0.600 ± 0.0001 ^a^	0.429 ± 0.0488 ^c^	0.414 ± 0.0378 ^c^	0.543 ± 0.0535 ^b^	0.457 ± 0.0535 ^c^
Adhesiveness (mJ)	0.663 ± 0.124 ^a^	0.256 ± 0.149 ^c^	0.476 ± 0.0441 ^b^	0.281 ± 0.176 ^c^	0.339 ± 0.195 ^bc^	0.394 ± 0.186 ^bc^
Rupture force (N)	43.8 + 3.20 ^a^	25.5 + 0.704 ^b^	14.7 + 1.43 ^e^	10.7 + 1.41 ^f^	22.1 + 2.35 ^c^	18.1 + 1.03 ^d^
Shear stress (N)	38.0 ± 2.89 ^a^	27.8 ± 7.58 ^b^	29.5 ± 5.09 ^b^	20.4 ± 3.51 ^c^	26.9 ± 0.786 ^bc^	25.2 ± 1.45 ^bc^
a_w_	0.978 ± 0.569 ^c^	0.984 ± 0.267 ^b^	0.988 ± 0.101 ^ab^	0.987 ± 0.132 ^ab^	0.99 ± 0.110 ^a^	0.99 ± 0.070 ^a^
Processing yield (%)	94.5 ± 0.467 ^bc^	92.4 ± 0.0564 ^d^	95.0 ± 0.0505 ^a^	91.9 ± 0.0541 ^e^	94.9 ± 0.104 ^ab^	94.3 ± 0.0594 ^c^
CL	23.2 ± 0.324 ^d^	25.5 ± 0.559 ^c^	28.0 ± 0.4170 ^b^	30.2 ± 0.164 ^a^	28.6 ± 0.660 ^b^	30.6 ± 0.241 ^a^
L*	43.1 ± 0.551 ^c^	58.9 ± 0.203 ^b^	59.6 ± 0.0666 ^a^	58.7 ± 0.356 ^b^	59.6 ± 0.214 ^a^	59.7 ± 0.459 ^a^
a*	16.1 ± 0.733 ^c^	17.5 ± 0.250 ^ab^	17.1 ± 0.247 ^ab^	17.7 ± 0.156 ^a^	17.4 ± 0.108 ^ab^	16.9 ± 0.197 ^b^
b*	11.4 ± 0.269 ^a^	9.07 ± 0.174 ^d^	10.8 ± 0.0458 ^b^	10.5 ± 0.146 ^b^	9.92 ± 0.246 ^c^	10.3 ± 0.549 ^bc^
Chroma	19.8 ± 0.650 ^b^	19.7 ± 0.274 ^b^	20.2 ± 0.231 ^ab^	20.6 ± 0.134 ^a^	20.0 ± 0.211 ^ab^	19.8 ± 0.356 ^b^
Hue°	35.3 ± 1.260 ^a^	27.3 ± 0.405 ^d^	32.1 ± 0.275 ^b^	30.7 ± 0.465 ^bc^	29.7 ± 0.477 ^c^	31.2 ± 1.330 ^b^

Values represent the means ± SD. Different letters in the same row indicate statistical differences (*p* < 0.05) among sausages. C1: high-fat Frankfurter-type sausage formulation; C2: low-fat Frankfurter-type sausage with carrageenan formulation; F1: dragon fruit peel powder (DFPP) formulation; F2: insoluble dietary fiber (IDF) formulation; F3: soluble dietary fiber (SDF) formulation; and F4: mix of IDF/SDF (1/1) formulation. L*: Lightness scale (0 = black and 100 = white); a*: Red/green coordinates (positive values indicate redness and negative values indicate greenness); b*: Yellow/blue coordinates (positive values indicate yellowness and negative values indicate blueness); chroma: indicates the purity of color (saturation), hue°: is the angular representation of color.

**Table 8:** Chemical composition of the best formulation (F3) with fat replacement by soluble dietary fiber (SDF) obtained from dragon fruit peel powder (DFPP) in an alpaca-based sausage.

Chemical Composition	Amount *
Moisture (%)	70.6 ± 0.021
Protein (%)	14.0 ± 0.120
Fat (%)	4.01 ± 0.002
Total carbohydrates (%)	8.53 ± 0.136
Ash (%)	2.91 ± 0.035
Crude fiber (%)	0.30 ± 0.028
Iron (ppm)	19.0 ± 0.007
Zinc (ppm)	11.9 ± 0.078

* Values are presented as means ± SD (*n* = 3).

## Data Availability

The data presented in this study are available on request from the corresponding author.

## Supplementary Materials

The following supporting information can be downloaded at: https://www.mdpi.com/article/10.3390/foods12152945/s1, Figure S1: Illustration of yellow dragon fruit (*Hylocereus megalanthus*): (**a**) whole fruit, (**b**) peel separation from fruit, and (**c**) peel only. (Scale bar = 2.5 cm). Table S1: Mathematical models used to describe the drying kinetics of yellow dragon fruit peel. Table S2: Statistical fitting parameters (RMSE, RSE, R^2^ and MAPE) of the mathematical models analyzed at different temperatures. Table S3: The coefficients of the mathematical models (a, b, c, n, k_0_ or k_1_) analyzed at three different temperatures (40, 55 and 70 °C). Table S4: Summary of statistics of analysis of variance (ANOVA) for reduced quadratic models for insoluble dietary fiber (IDF), soluble dietary fiber (SDF), and degree of esterification (DE).

## Abbreviations

RMSE: root mean square error; RSE: relative squared error; R^2^: coefficient of determination; MAPE: mean absolute percentage error; SD: standard deviation; XR: moisture ratio; D_eff_: effective moisture diffusivity; IDF: Insoluble dietary fiber; SDF: soluble dietary fiber; YDFP: yellow dragon fruit peels; DFPP: dragon fruit peel powder; DE: degree of esterification; TUAT: total ultrasound application time; PT: pause time; LSR: liquid-to-solid ratio; WHC: water-holding capacity; OHC: oil-holding capacity; C1: high-fat Frankfurter-type sausage formulation; C2: low-fat Frankfurter-type sausage with carrageenan formulation; F1: dragon fruit peel powder formulation; F2: insoluble dietary fiber formulation; F3: soluble dietary fiber formulation; F4: mix of IDF and SDF (1/1) formulation; SC: swelling capacity; CP: commercial pectin; TPA: texture profile analysis; CL: cooking losses; a_w_: water activity; PY: processing yield; L*: lightness; a*: redness; b*: yellowness; 3 D: three-dimensional.
