# Supplementary material for: Optimization of Ultrasound-Assisted Extraction of Dietary Fiber from Yellow Dragon Fruit Peels and Its Application in Low-Fat Alpaca-Based Sausages
Source: Foods. 2023 Aug 3;12(15):2945. doi: 10.3390/foods12152945 (PMC10419239; doi:10.3390/foods12152945)
Supplement: Supplementary file 1 [file foods-12-02945-s001.zip › foods-2499208-supplementary.pdf]

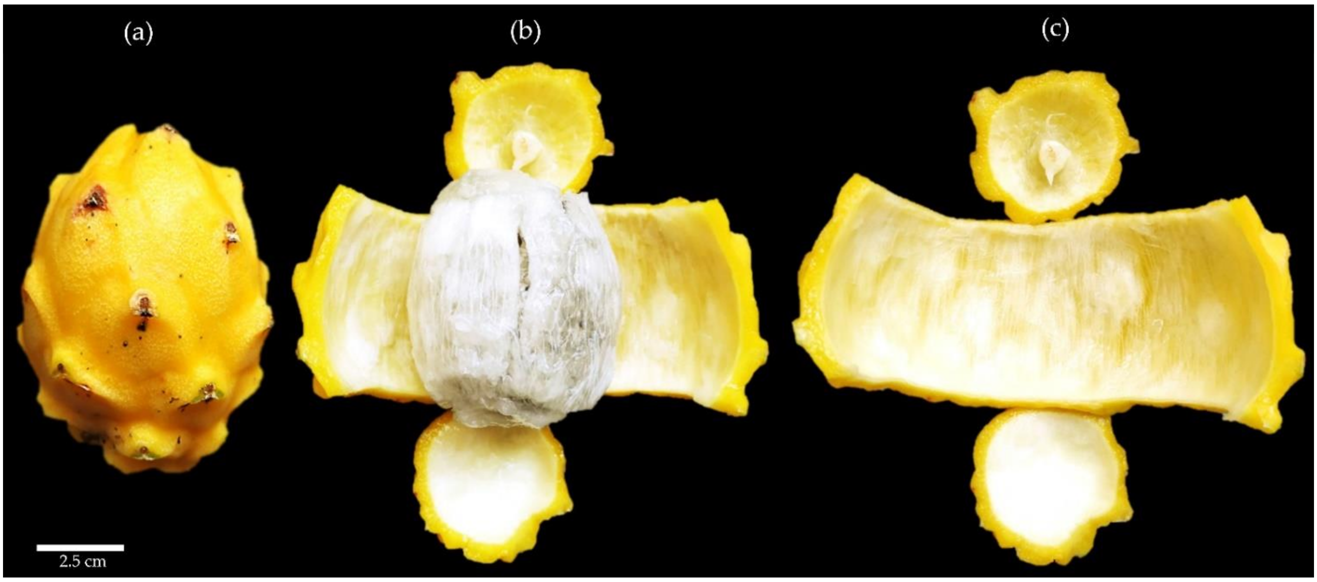

Figure S1. Illustration of yellow dragon fruit (*Hylocereus megalanthus*): (a) whole fruit, (b) peel separation from fruit, and (c) peel only. (Scale bar = 2.5 cm).

Table S1. Mathematical models used to describe the drying kinetics of yellow dragon fruit peel.

Model name	Model
Peleg	$XR = 1 - (t/(k_0 + k_1*t))$
Double Exponential term	$XR = a * \exp(-k_0*t) + (1 - a) * \exp(-k_0*a*t)$
Newton	$XR = \exp(-k_0*t)$
Henderson & Pabis	$XR = a*\exp(-k_0*t)$
Logarithmic	$XR = a*\exp(-k_0*t) + c$
Double term	$XR = a*\exp(-k_0*t) + b*\exp(-k_1*t)$
Aghbashlo	$XR = \exp(-((a*t)/(1+b*t)))$
Page	$XR = \exp(-k_0*t\verb|^|n)$
Modified Page	$XR = \exp(-(k_0*t)\verb|^|n)$
Weibull	$XR = \exp(-(t/b)\verb|^|a)$
Midilli	$XR = a*\exp(-k_0*t\verb|^|n) + b*t$

XR: moisture ratio (dimensionless); t: time (h); k_0, and k_1: drying rate constant (h^{-1}); a, b, c, and n: drying kinetic constants.

Table S2. Statistical fitting parameters (RMSE, RSE, R^2 and MAPE) of the mathematical models analyzed at different temperatures.

Temperature (°C)	Model	RMSE (x 10^{-03} ± SD x 10^{-04})	RSE (x 10^{-04} ± SD x 10^{-05})	R^2 (SD x 10^{-05})	MAPE
	Aghbashlo	2.79 ± 2.11^{f}	1.18 ± 1.59^{d}	0.9999 ± 1.25^{a}	1.05 ± 0.474^{b}
	Double term	2.63 ± 1.80^{f}	1.05 ± 1.56^{d}	0.9999 ± 1.52^{a}	0.57 ± 0.417^{b}
	Two-term exponential	3.35 ± 5.51^{ef}	1.73 ± 5.13^{d}	0.9999 ± 3.94^{a}	2.03 ± 0.835^{b}
	Henderson & Pabis	8.17 ± 3.06^{c}	10.1 ± 5.99^{c}	0.9993 ± 3.52^{b}	3.21 ± 1.29^{b}
	Logarithmic	4.30 ± 2.83^{d}	2.80 ± 4.06^{d}	0.9997 ± 4.06^{ab}	1.73 ± 1.16^{b}
40	Midilli	1.84 ± 2.23^{g}	0.51 ± 1.15^{d}	0.9999 ± 1.15^{a}	0.63 ± 0.314^{b}
	Newton	10.6 ± 4.48^{b}	17.1 ± 11.9^{b}	0.9997 ± 2.40^{ab}	3.61 ± 1.43^{b}
	Page	3.59 ± 4.59^{de}	1.97 ± 4.59^{d}	0.9999 ± 3.59^{a}	2.09 ± 0.861^{b}
	Modified Page	3.59 ± 4.59^{de}	1.97 ± 4.59^{d}	0.9999 ± 3.59^{a}	2.09 ± 0.861^{b}
	Peleg	28.9 ± 9.48^{a}	127 ± 103^{a}	0.9886 ± 97.6^{c}	15.7 ± 9.14^{a}
	Weibull	3.59 ± 4.59^{de}	1.97 ± 4.59^{d}	0.9999 ± 3.59^{a}	2.09 ± 0.862^{b}
	Aghbashlo	4.42 ± 3.52^{de}	2.75 ± 4.15^{d}	0.9998 ± 3.88^{ab}	0.46 ± 0.342^{b}
	Double term	6.78 ± 29.2^{d}	7.34 ± 54.1^{d}	0.9995 ± 38.8^{ab}	1.63 ± 0.909^{b}
	Two-term exponential	3.12 ± 10.8^{e}	1.45 ± 9.23^{d}	0.9999 ± 6.37^{a}	1.41 ± 1.43^{b}
	Henderson & Pabis	11.3 ± 28.9^{c}	18.3 ± 84.4^{c}	0.9987 ± 59.9^{c}	2.63 ± 2.54^{b}
	Logarithmic	7.22 ± 11.9^{d}	7.37 ± 20.8^{d}	0.9993 ± 20.8^{b}	1.98 ± 2.07^{b}
55	Midilli	1.87 ± 4.34^{e}	0.50 ± 2.09^{d}	0.9999 ± 2.09^{a}	0.33 ± 0.304^{b}
	Newton	15.5 ± 34.9^{b}	34.2 ± 138^{b}	0.9992 ± 28.6^{bc}	3.13 ± 3.00^{b}
	Page	2.90 ± 11.7^{e}	1.28 ± 9.40^{d}	0.9999 ± 7.32^{a}	1.32 ± 1.28^{b}
	Modified Page	2.90 ± 11.7^{e}	1.28 ± 9.40^{d}	0.9999 ± 7.32^{a}	1.32 ± 1.28^{b}
	Peleg	31.3 ± 3.50^{a}	137 ± 92.1^{a}	0.9877 ± 86.9^{d}	11.5 ± 8.80^{a}
	Weibull	2.90 ± 11.7^{e}	1.28 ± 9.40^{d}	0.9999 ± 7.32^{a}	1.32 ± 1.28^{b}
	Aghbashlo	5.17 ± 16.0^{d}	4.56 ± 24.4^{cd}	0.9996 ± 22.0^{abc}	0.43 ± 0.038^{b}
	Double term	8.66 ± 17.8^{c}	12.5 ± 42.7^{cd}	0.9989 ± 38.1^{abc}	1.15 ± 0.584^{b}
	Two-term exponential	2.31 ± 6.11^{d}	0.90 ± 4.07^{d}	0.9999 ± 4.04^{a}	0.60 ± 0.248^{b}
	Henderson & Pabis	10.2 ± 18.5^{c}	17.2 ± 50.8^{bc}	0.9985 ± 44.8^{c}	1.25 ± 0.637^{b}
	Logarithmic	8.93 ± 16.1^{c}	13.3 ± 38.6^{cd}	0.9987 ± 38.6^{abc}	2.40 ± 1.89^{b}
70	Midilli	2.57 ± 6.83^{d}	1.11 ± 5.06^{d}	0.9999 ± 5.04^{ab}	0.47 ± 0.057^{b}
	Newton	13.3 ± 24.0^{b}	29.4 ± 86.1^{b}	0.9986 ± 37.9^{bc}	1.51 ± 0.752^{b}
	Page	2.74 ± 7.18^{d}	1.27 ± 5.67^{d}	0.9999 ± 5.49^{ab}	0.56 ± 0.230^{b}
	Modified Page	2.74 ± 7.18^{d}	1.27 ± 5.67^{d}	0.9999 ± 5.49^{ab}	0.56 ± 0.230^{b}
	Peleg	39.2 ± 0.52^{a}	255 ± 176^{a}	0.9766 ± 171^{d}	19.5 ± 15.8^{a}
	Weibull	2.74 ± 7.18^{d}	1.27 ± 5.67^{d}	0.9999 ± 5.49^{ab}	0.56 ± 0.230^{b}

Similar lowercase letters indicate no significant ($p > 0.05$) differences for comparisons between the different mathematical models in columns for each temperature (40, 55 or 70 °C). Fitting statistics: Root mean squared error (RMSE), relative squared error (RSE), coefficient of determination (R^2), and mean absolute percent error (MAPE) are also shown.

Table S3. The coefficients of the mathematical models (a, b, c, n, k_0 or k_1) analyzed at three different temperatures (40, 55 and 70 °C).

Model	Coefficients	Temperature (°C)		
		40	55	70
Double term	a	2.7647	4.7604	-0.3897
	b	-1.7517	-3.7558	1.4163
	k_0	0.3887	0.9963	0.6084
	k_1	0.3413	1.1055	0.8771
Two-term exponential	a	1.5215	1.6034	1.6107
	k_0	0.5827	0.8877	1.2510
Henderson & Pabis	a	1.0297	1.0430	1.0400
	k_0	0.5016	0.7400	1.0355
Logarithmic	a	1.0325	1.0478	1.0418
	c	-1.26×10^{-02}	-1.63×10^{-02}	-6.82×10^{-03}
	k_0	0.4801	0.7009	1.0111
Newton	k_0	0.4876	0.7107	0.9972
Page	n	1.0752	1.1112	1.1128
	k_0	0.4540	0.6675	0.9726
Modified Page	n	1.0752	1.1112	1.1128
	k_0	0.4798	0.6950	0.9753
Peleg	k_0	1.5893	1.1330	0.7138
	k_1	0.8064	0.7877	0.8474
Midilli	a	0.9983	0.9994	0.9946
	b	-6.33×10^{-04}	-7.42×10^{-04}	-1.25×10^{-05}
	n	1.0637	1.1014	1.1208
	k_0	0.4540	0.6660	0.9654
Weibull	a	1.0752	1.1112	1.1128
	b	2.0841	1.4388	1.0253
Aghbashlo	a	0.4486	0.6324	0.8901
	b	-2.63×10^{-02}	-5.34×10^{-02}	-0.0735

k_0 and, k_1: drying rate constant (h^{-1}); a, b, c, n: drying kinetic constants.

Table S4. Summary of statistics of analysis of variance (ANOVA) for reduced quadratic models for insoluble dietary fiber (IDF), soluble dietary fiber (SDF), and degree of esterification (DE).

Dependent Variables	ANOVA											Goodness-of-fit				
	Model	PT	LSR	TUAT	PT x LSR	PT x TUAT	LSR x TUAT	PT^2	LSR^2	$TUAT^2$	Lack of Fit	CV (%)	PRESS (-)	R^2	R^2 adj	R^2 pred
IDF (%)	19.7 (0.0009)	4.56 (0.0766)	88.3 (<0.0001)	17.3 (0.0059)	8.12 (0.0292)	12.1 (0.0132)	—	5.80 (0.0527)	18.3 (0.0052)	6.99 (0.0384)	0.334 (0.8394)	2.94	84.8	0.9634	0.9145	0.7867
SDF (%)	23.0 (0.0003)	24.9 (0.0016)	21.0 (0.0025)	43.9 (0.0003)	8.88 (0.0205)	19.5 (0.0031)	—	11.7 (0.0111)	33.5 (0.0007)	—	1.61 (0.4260)	4.60	7.98	0.9583	0.9165	0.7137
DE (%)	87.1 (<0.0001)	343 (<0.0001)	29.8 (0.0016)	211 (<0.0001)	7.05 (0.0378)	—	14.4 (0.0090)	15.7 (0.0074)	19.4 (0.0046)	49.0 (0.0004)	1.21 (0.4998)	1.33	19.7	0.9915	0.9801	0.9341

F-test, *p*-value (in parentheses) and lack-of-fit from polynomial models (including: main, interaction, and quadratic terms) fitted on the insoluble dietary fiber (IDF), soluble dietary fiber (SDF) and degree of esterification (DE). "—" : Term dropped from analysis due to non-statistical significance ($p > 0.05$). Measurements of goodness-of-fit were: CV (coefficient of variation), PRESS (predicted residual error sum of squares), R^2 (coefficient of determination), R^2 adj (R^2 adjusted), and R^2 pred (R^2 predicted).
